# Progress on Exploring the Luminescent Properties of Organic Molecular Aggregates by Multiscale Modeling

**DOI:** 10.3389/fchem.2021.808957

**Published:** 2022-01-12

**Authors:** Jingyi Zhao, Xiaoyan Zheng

**Affiliations:** Beijing Key Laboratory of Photoelectronic/Electrophotonic Conversion Materials, Key Laboratory of Cluster Science of Ministry of Education, Key Laboratory of Medical Molecule Science and Pharmaceutics Engineering, Ministry of Industry and Information Technology, School of Chemistry and Chemical Engineering, Beijing Institute of Technology, Beijing, China

**Keywords:** aggregation-induced emission, structure–property relationship, non-covalent interactions, aggregation effect, multiscale modeling

## Abstract

Luminescent molecular aggregates have attracted worldwide attention because of their potential applications in many fields. The luminescent properties of organic aggregates are complicated and highly morphology-dependent, unraveling the intrinsic mechanism behind is urgent. This review summarizes recent works on investigating the structure–property relationships of organic molecular aggregates at different environments, including crystal, cocrystal, amorphous aggregate, and doped systems by multiscale modeling protocol. We aim to explore the influence of intermolecular non-covalent interactions on molecular packing and their photophysical properties and then pave the effective way to design, synthesize, and develop advanced organic luminescent materials.

## Introduction

In recent years, organic luminescent materials have attracted considerable attention due to their practical applications in optoelectronic devices, such as organic light-emitting diodes (OLEDs), organic light-emitting transistors (OLETs), and sensors (Ostroverkhova, 2016; Zhao Z. et al., 2018; Salehi et al., 2019). Traditional organic luminescence always shows bright emission in solution, but weak or quenched emission in aggregated states, that is the aggregation-caused quenching (ACQ) effect (Watson and Livingston, 1948). However, organic luminescent materials are usually used in aggregated states. The notorious ACQ effect has significantly hindered the development of organic luminescence. Fortunately, Tang’s group proposed the aggregation-induced emission (AIE) concept: organic molecules exhibit weak emission or non-emission in dilute solutions, while emitting brightly in the aggregated states (Luo et al., 2001; Tang et al., 2001). A large number of AIE luminogens (AIEgens) have been designed and synthesized in recent years, and it opens an avenue to an array of possibilities for their applications in photoelectric (Furue et al., 2016; Liu et al., 2020), medical (Song et al., 2020; Sharath Kumar et al., 2021), environmental (Cheng et al., 2017; Wang et al., 2020), and military (Zhou et al., 2019) fields and so on (Mei et al., 2015). It is demonstrated that molecular aggregates usually show different photophysical properties from dispersed monomers in dilute solutions, and their luminescent properties are usually highly morphology-dependent. For example, Mutai et al. (2014) found that 6-Cyano 2-(2′-Hydroxyphenyl)imidazo[1,2-a]pyridine has three different crystals but emits three different fluorescent colors due to their divergent molecular packing. Tang et al. showed that some nitro-substituted tetraphenylethylene (TPE) and triphenylamine (TPA) are non-emissive in the crystal state but glitter brightly in amorphous aggregates (Zhao W. et al., 2018). Zhao et al. proposed a series of organic emitters by integrating planar and distorted functional groups (donor, acceptor, or π-plane) with long alkyl side chains, which could impart bright emission in both solution and solid states (Wu et al., 2019). In addition, introducing a supramolecular host molecule into the AIEgen can effectively enhance the emission efficiency in both the monomer and aggregated states due to the non-covalent interactions between the host and guest molecules (Liang et al., 2014; Song et al., 2014; Liang et al., 2016; Liow et al., 2017). And, doping trace amounts of luminophores into host molecules makes efficient room temperature phosphorescence (Hirata et al., 2013; Kabe and Adachi, 2017; Han et al., 2019; Lei et al., 2019; Lei et al., 2021; Xie et al., 2021). Therefore, the luminescent properties of organic molecular aggregates are sensitive to molecular packing and intermolecular non-covalent interactions and are highly complicated.

Exploring the structure–property relationships of organic molecular aggregates is of great importance in designing, synthesizing, and developing advanced luminescent candidates. In experiments, the restriction of intramolecular rotation (RIR) mechanism, the intramolecular vibration (RIV) mechanism, and also the restriction of motion (RIM) mechanism were proposed to explain the AIE phenomenon (Chen et al., 2003; Dong et al., 2007; Hong et al., 2009; Leung et al., 2014; Mei et al., 2014; Tu et al., 2021). Theoretically, Peng and Shuai et al. proposed that the trip-out of electron–vibration coupling blocks the excited-state non-radiative decay channels in aggregated states and turns fluorescence on (Peng et al., 2007a; Peng et al., 2007b; Niu et al., 2010). Li and Blancafort et al. put forward the restricted access to a conical interaction (RACI) mechanism based on the potential energy surface analysis (Peng et al., 2016; Crespo-Otero et al., 2019). Shuai’s group also proposed the blockage of non-radiative decay *via* the minimum energy crossing point (MECP) away from the harmonic region in aggregates (Ou et al., 2020; Peng and Shuai, 2021). In addition, other scenarios have also been declared, including excited-state intramolecular proton-transfer (ESIPT)–inspired solid state emitters (Padalkar and Seki, 2016; Zhao J. et al., 2019), the restriction of the E/Z isomerization mechanism (Chung et al., 2013), the blockage of access to the dark state with n → π* or σ→ π* in the aggregation phase (Ma et al., 2016; Tu et al., 2019; Peng et al., 2021; Tu et al., 2021), halogen bonding interactions–induced effective phosphorescence (Cai et al., 2018; Yang et al., 2020), the energy transfer–facilitated room temperature phosphorescence in a trace amount guest-doped host-matrix system (Lei et al., 2020; Lei et al., 2021; Wang et al., 2021), and so on.

**FIGURE 1:**
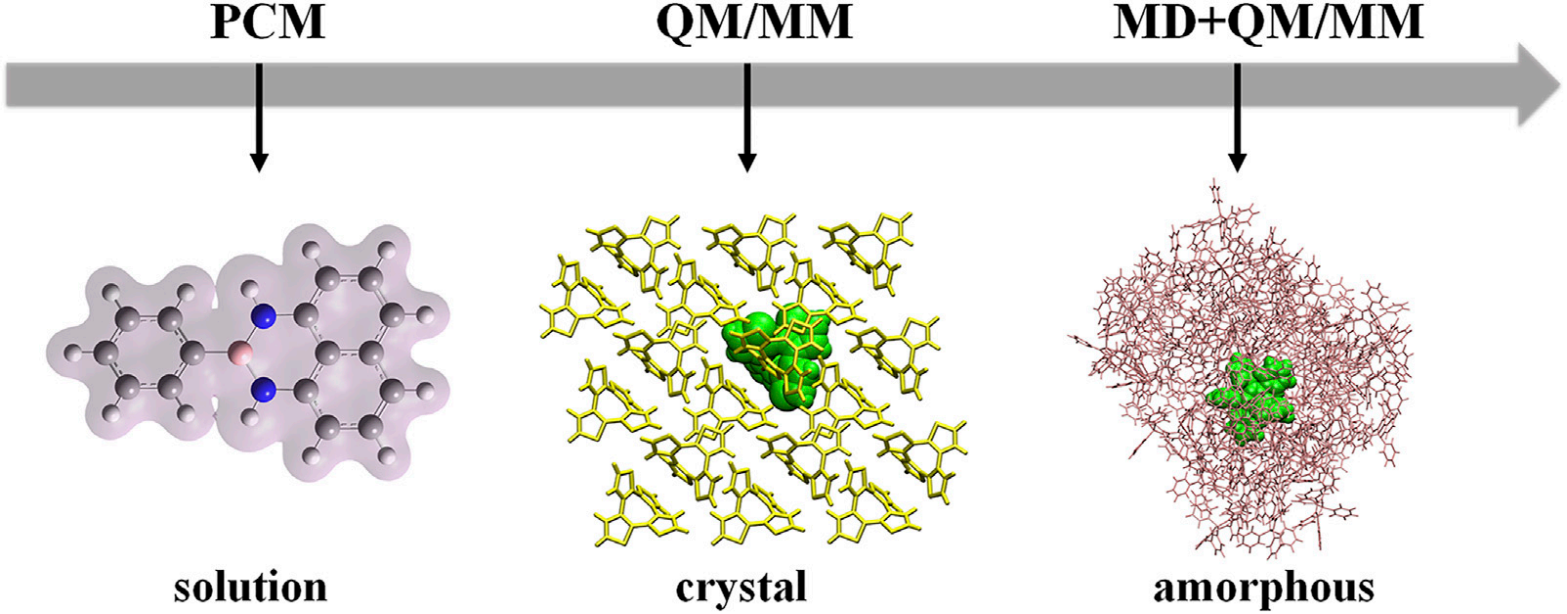
Setup models of organic molecules in dilute solution, crystal, and amorphous aggregates.

Theoretical calculations play key roles in exploring the relationships between molecular structures and luminescent properties. The luminescent properties of AIEgens are highly environment-dependent, so different molecular models need to be setup according to the relevant environments in experiments, such as the dilute solution, amorphous aggregate, and crystal (Figure 1). It is well-known that most AIE phenomena are usually confirmed in the solution, so the model setup should consider the solvent effect. Especially, molecules with intramolecular charge transfer properties are quite sensitive to the solvent polarity, and they usually demonstrate red-shifted emission as polarity increases (Tu et al., 2020; Zhang et al., 2020). The implicit solvation models, such as the polarizable continuum model (PCM), are good for considering the solvent effect (Tomasi et al., 2005; Fan et al., 2015; Provorse Long and Isborn, 2017). For the solvent insensitive molecules, to be simple, sometimes the model is setup at the gas phase directly. For the solid phase, we build the hybrid quantum mechanics/molecular mechanics (QM/MM) model to consider the influence of molecular packing on photophysical properties of the studied system (Vreven et al., 2006; Lior-Hoffmann et al., 2012; Van der Kamp and Mulholland, 2013; Chung et al., 2015; Shen et al., 2016; Pahima et al., 2019). During the QM/MM calculations, the QM molecule is active, and all other molecules in the MM region are frozen. The density functional theory (DFT)/time-dependent DFT (TDDFT) are chosen to deal with the luminescent properties of the QM molecule at the ground and excited states, respectively. For crystal, the QM/MM model can be setup based on the experimental X-ray crystal structure. However, the molecular conformations of AIEgens in amorphous aggregates or thin films are not available in experiments. The large timescale molecular dynamics (MD) simulations need to be performed to determine the conformations (Zheng et al., 2019). Then, the QM/MM models are setup, and the photophysical properties of AIEgens are calculated based on the obtained MD conformations accordingly. Moreover, the molecular packing of amorphous aggregates is irregular, resulting in distinct local environments for each molecule in the amorphous aggregate. It is noted that we need to consider various molecular packings in amorphous aggregates during calculations.

In this review, we will summarize some recent works to demonstrate the relationship between molecular packing of organic molecules and their luminescent properties at the atomic level by using multiscale modeling protocol. Here, the influence of intermolecular non-covalent interactions on the molecular packing and their photophysical properties are highlighted at several representative environments, including from the regular packing crystal to the irregular amorphous aggregates and then from the host–guest complexation by supramolecular self-assembly to the solvent-involved cocrystal, and then to host–guest doping systems. This work emphasizes the ability of multiscale modeling protocol in explaining the luminescent properties of organic molecular aggregates.

## Luminescent Properties of Organic Aggregates

### Organic Aggregates of the Propeller-Shaped Silole System

**SCHEME 1:**
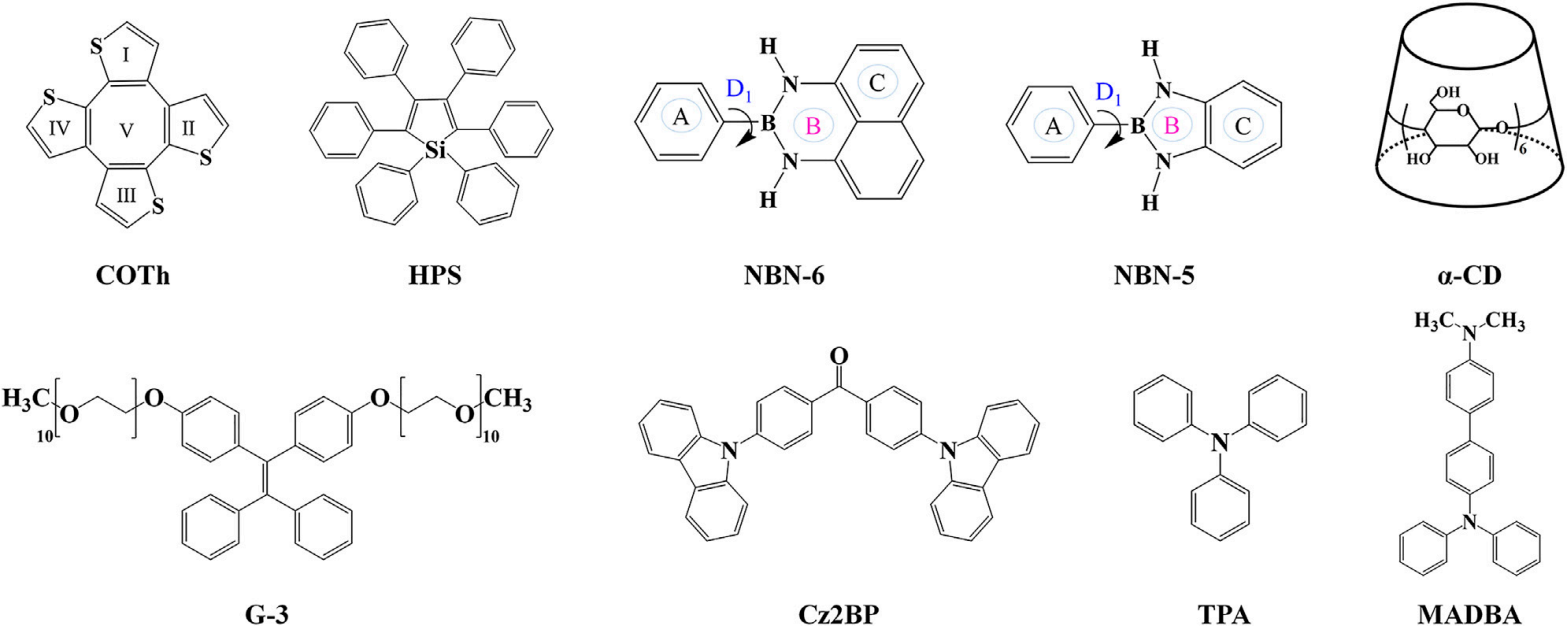
Overview of molecular structures discussed in this review.

**FIGURE 2:**
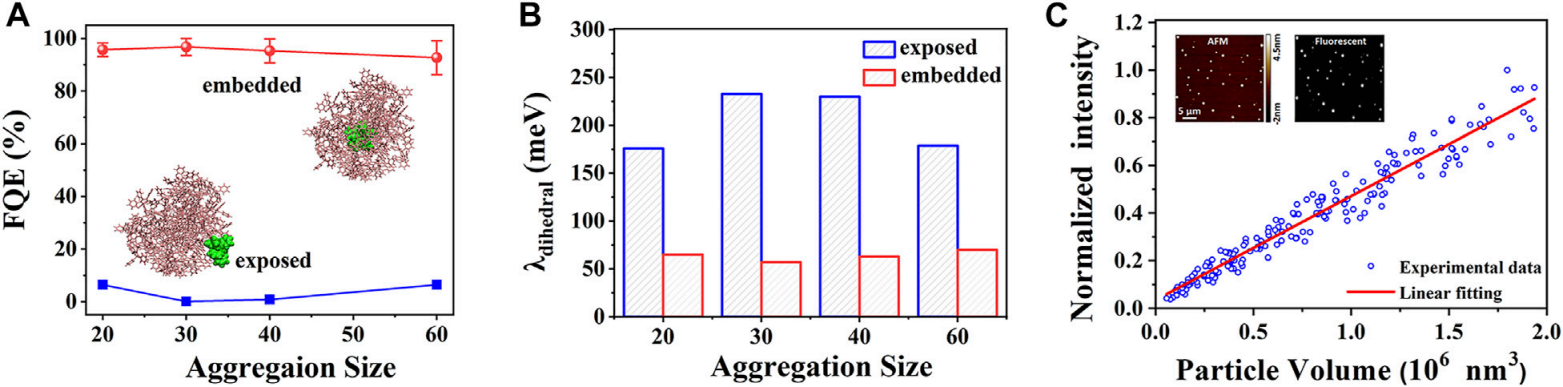
**(A)** Average fluorescence quantum efficiency (FQE) of four embedded (red) and exposed (blue) HPS molecules extracted from amorphous aggregates with different sizes, and the representative embedded and exposed QM/MM models of HPS are shown in the insets. **(B)** *λ*_dihedral_ of embedded and exposed HPS molecules in different sizes of aggregates. **(C)** Fluorescent intensity is linear to the volume of HPS aggregates. The volume (inset, atomic force microscope (AFM) image) and fluorescence (inset, fluorescence image) of individual HPS aggregate measured by fluorescence confocal atomic force microscopy. The red solid line is the linear fitting of experimental data (Jiang et al., 2017). (Reproduced with permission from Zheng et al. (2016); Copyright 2016 The Royal Society of Chemistry).

The first molecule discussed here belongs to the typical propeller-shaped AIE system. Unlike crystals with periodic molecular packing, amorphous aggregates are structurally heterogeneous; it is a great challenge to investigate the aggregation effects on photophysical properties of AIEgens. Taking the emblematic hexaphenylsilole (HPS) as an example (see Scheme 1), the aggregation effect of HPS was systematically investigated by simulating four different sizes of amorphous aggregates (20, 30, 40, and 60) by combining MD and QM/MM calculations (Zheng et al., 2016). The embedded QM/MM model and exposed QM/MM model (insets in Figure 2A) were setup, respectively, to differentiate the different environments of HPS embedded inside the amorphous aggregate and exposed on the surface. In addition, for each aggregate size, five embedded molecules with different conformations were selected, and their photophysical properties were calculated accordingly to include the impact of the molecular packing difference. Compared to HPS crystal, the fluorescent emission in amorphous aggregate is red-shifted, giving a direct interpretation for the crystallization-enhanced emission phenomenon in the experiment (Dong et al., 2015; Zhao et al., 2020). The fluorescence quantum efficiency (FQE) was calculated by *η*_F_≈*k*_r_/(*k*_r_ + *k*_ic_), where *k*_r_ is the radiative decay rate constant, and *k*_ic_ is the non-radiative decay rate constant, respectively. The FQE of both the embedded (>92.7%) and exposed HPS molecules (<7%) are size-independent, and the FQE of embedded HPS is 1–2 orders of magnitude larger than those of the exposed ones (Figure 2A). This is because the environment-insensitive *k*_r_ is hardly changed, but the *k*_ic_ of embedded HPS molecules are significantly smaller than those of exposed ones. Analyzing the key parameter of reorganization energy (*λ*) determining *k*_ic_, it indicates that the *λ* of dihedral angles (*λ*_dihedral_) mainly contributes to the different *k*_ic_ between the embedded and the exposed HPS molecules in amorphous aggregates (Figure 2B), implying that the densely packed amorphous aggregate significantly blocks the non-radiative decay channel of the excited state energy by retarding the electron–vibration coupling of the low-frequency rotational modes of phenyl rings in HPS. We conclude that the FQE of the nano-sized aggregate is size-independent, and the embedded molecules dominate their luminescent intensity; therefore we predict there is a linear relationship between the fluorescent intensity and aggregate size, which was also successfully confirmed in the experiment (Jiang et al., 2017; Figure 2C).

### Organic Aggregates of the Annulene-Based System Without Rotors

**FIGURE 3:**
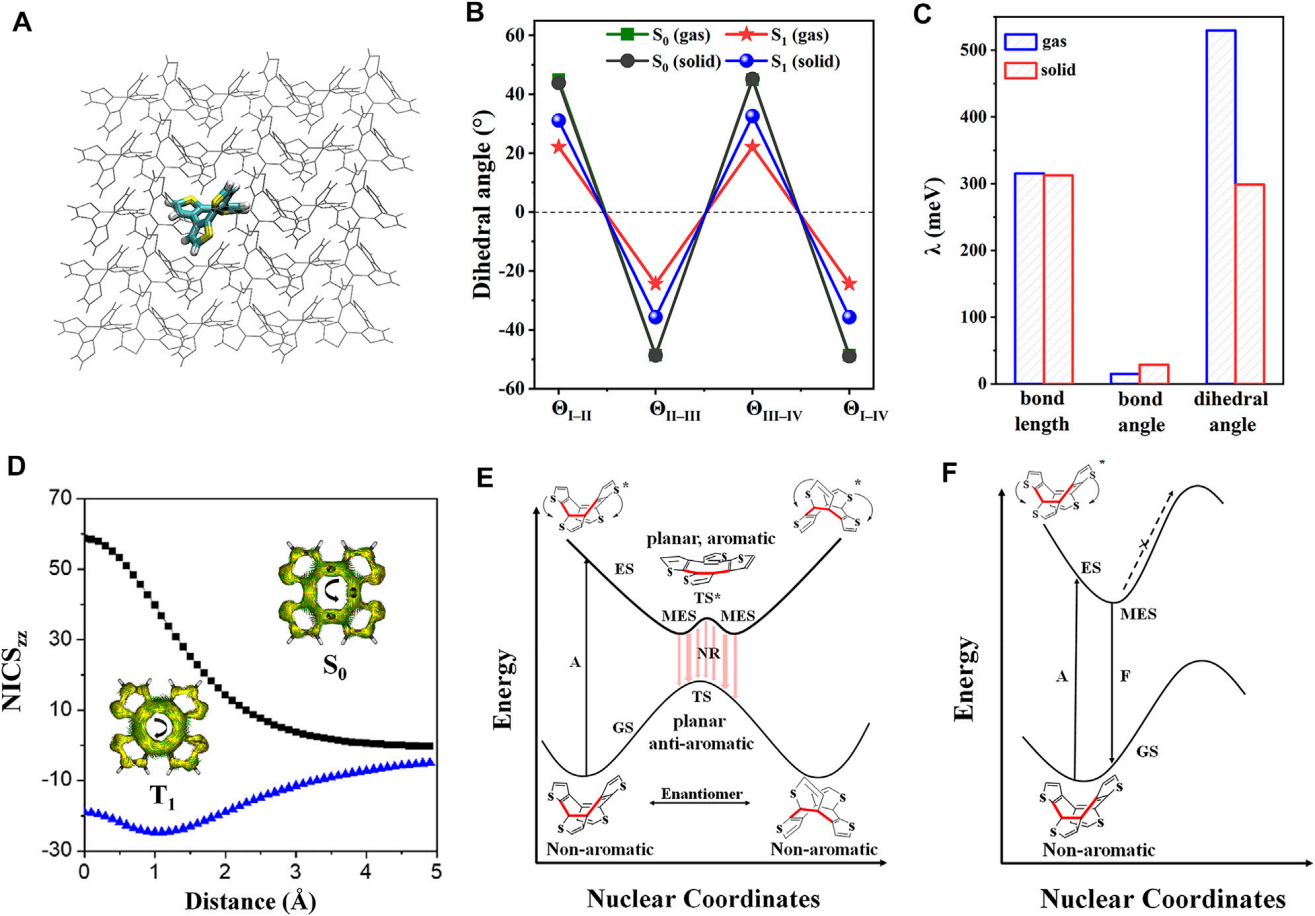
**(A)** QM/MM model of COTh in the crystalline state. **(B)** Key dihedral angles in both dilute solution and crystal at S_0_ and S_1_ states. **(C)** Projection *λ* of COTh on internal coordinates, including bond lengths, bond angles, and dihedral angles. **(D)** NICS_zz_ scan of COTh based on its transition-state structures at the S_0_ (black) and T_1_ (blue) states. The ACID plots of both S_0_ and T_1_ states were in the inset. Proposed decay pathways along the potential energy surface of COTh **(E)** in dilute solution and **(F)** in solid state. Abbreviation: GS, ground state; ES, excited state; TS, transition state; MES, minimum energy structure; A, absorption; F, fluorescence; NR, non-radiative decay. (Reproduced with permission from (Zhao Z. et al., 2019); This figure is extracted from an open access journal with thanks; Copyright 2019 Nature Publishing Group).

Cyclooctatetrathiophene (COTh, Scheme 1) is a non-aromatic annulene-based eight-member ring (Zhao J. et al., 2019); it demonstrates aromaticity reversal property upon excitation, following the Baird’s rule that the aromatic (anti-aromatic) molecule at the ground state (S_0_) reverses to the anti-aromatic (aromatic) property at the lowest excited triplet state (T_1_) (Baird, 1972). The aromaticity reversal can serve as a driving force inducing the significant conformational change to quench the emission. Thus, suppressing the excited-state aromaticity reversal of COTh turns the emission on (Zhao Z. et al., 2019). Theoretical calculations for COTh in both isolated and crystalline states were carried out to unravel the AIE mechanism at the atomic level. The isolated COTh was setup at the gas phase, and the crystalline state was simulated by the QM/MM model (Figure 3A) to consider the influence of molecular packing on its photophysical properties. Upon excitation, the dihedral angles of neighboring thiophene rings of COTh are dramatically changed; the eight-member ring became more planar (Figure 3B), while the corresponding bond lengths and bond angles are still similar. The change of dihedral angles upon irradiation in solution is more significant than those of the crystalline state, that is to say, the conformation of COTh in crystal is more constrained, supported by its smaller *λ* than that in solution. The larger *λ* in solution is mainly contributed by the change of dihedral angles (Figure 3C). In addition, the femtosecond transient-absorption spectra analysis also indicated that COTh has a very rapid molecular deformation in dilute solution, while the change is suppressed in the solid state.

The nucleus-independent chemical shift (NICS) and anisotropy of the induced current density (ACID) analysis were performed based on the S_0_ and T_1_ transition-state structures. As shown in Figure 3D, the highly positive NICS_zz_(1) at S_0_ and negative NICS_zz_(1) at T_1_ indicate COTh is anti-aromatic at S_0_ and aromatic at T_1_; therefore the aromaticity reversal occurred upon excitation in the COTh system. It is also supported by the different ring-current of the eight-member ring at S_0_ and T_1_ states (Figure 3D). In general, the COTh molecule adopts a tub-like conformation at S_0_ due to its non-aromatic feature (Liu et al., 2007; Nishinaga et al., 2010); it can suffer a quick conformational change to approach the planar/quasi-planar aromatic state (Kotani et al., 2020). Therefore, as illustrated in Figure 3E, there are two pathways for the isolated COTh molecule to stabilize the anti-aromaticity state at S_1_, go-up and go-down to reach the minimum energy structures (MES) corresponding to the non-radiative decay of exciton, resulting in the quenched emission. However, crystal densely packing effectively restricts the molecular deformation process and essentially enhances its emission (Figure 3F).

### Organic Aggregates of NBN-Doped Polycyclic Aromatic Hydrocarbons

**FIGURE 4:**
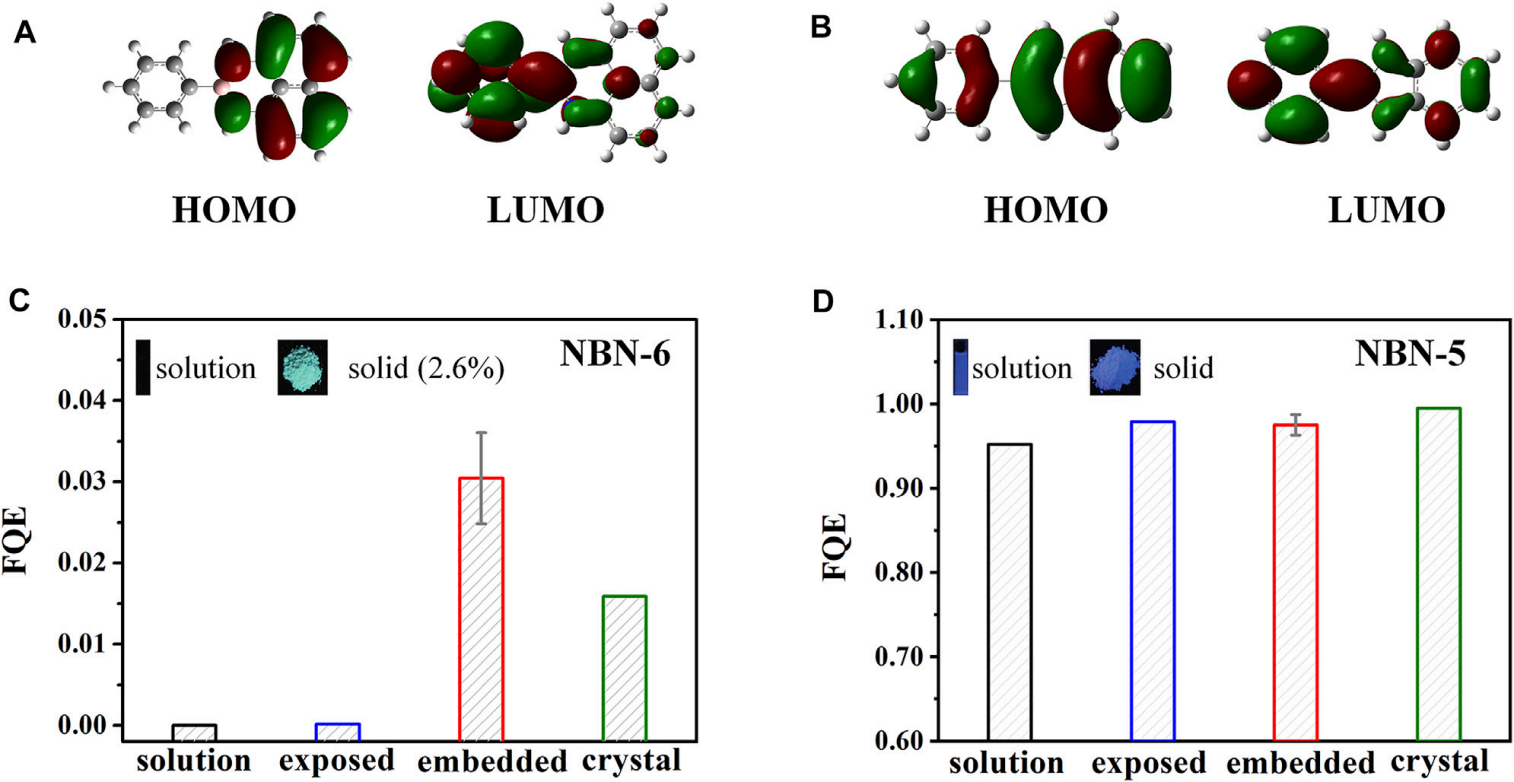
Electron density contours of HOMO and LUMO of **(A)** NBN-6 and **(B)** NBN-5 at S_0_ geometry in methanol solution. The FQE of **(C)** NBN-6 and **(D)** NBN-5 at different environments. (Reproduced with permission from (Zeng et al., 2021) Copyright 2021 The Royal Society of Chemistry).

Replacement of the C=C unit with its isoelectronic B–N unit can effectively alter the optoelectronic performances of polycyclic aromatic hydrocarbons (PAHs). NBN-5 and NBN-6 (Scheme 1) are two representative NBN-doped PAHs; NBN-6 is AIE-active, and NBN-5 could emit fluorescence in both solution and solid states (Wan et al., 2018). The photophysical properties of both compounds at different environments (including the dilute solution, amorphous aggregate, and crystal) were systematically explored by combining MD simulations and the thermal vibration correlation function–coupled QM/MM models (Zeng et al., 2021). The embedded and exposed QM/MM models were setup respectively (as discussed above) to consider the different molecular packing in amorphous aggregates. It is found that, upon excitation, the dihedral angle D_1_ between rings A and B of NBN-6 exhibits significant changes in both solution (27.0°) and exposed (16.0°) states, which are much larger than those of the embedded (about 5.1°) and crystalline (3.9°) state; in the meanwhile, the conjugation and planarity of NBN-6 at S_0_ are obviously improved after aggregation. Therefore, the rotation of ring A in NBN-6 can be effectively restricted in the aggregated state, leading to fluorescence enhancement. By contrast, the structural modifications of NBN-5 are pretty slight, with the largest structural change of D_1_ 5.7° in solution (much smaller than that of NBN-6). Furthermore, the configurations of NBN-5 are insensitive to environments, keeping rigid and planar structures in all cases. It is clear to see that NBN-6 has intramolecular charge transfer (ICT) property; the highest occupied molecular orbital (HOMO) is located on ring B and naphthalene moiety, while the lowest unoccupied molecular orbital (LUMO) is distributed on rings A and B (Figure 4A). In contrast, both the HOMO and LUMO of NBN-5 are delocalized on the whole backbone (Figure 4B). The ICT property of NBN-6 leads to their relatively lower energy gap and redder fluorescence emission at all environments than those of NBN-5. AIEgens with ICT properties are quite sensitive to the environments (Zhang et al., 2020); the FQE of NBN-6 is highly environment-dependent, with *k*_ic_ decreasing by 2–4 orders of magnitude after aggregation; thus NBN-6 is AIE-active (Figure 4C). Meanwhile, the FQE of NBN-5 is environment-independent, showing bright emission in both solution and solid states (Figure 4D). Normal mode analysis of NBN-6 at different environments indicates that the suppression of the out-of-plane rotation and distortion of ring A after aggregation is the primary reason for the AIE effect.

### Organic Aggregates Involving Supramolecular Host Molecules

**FIGURE 5:**
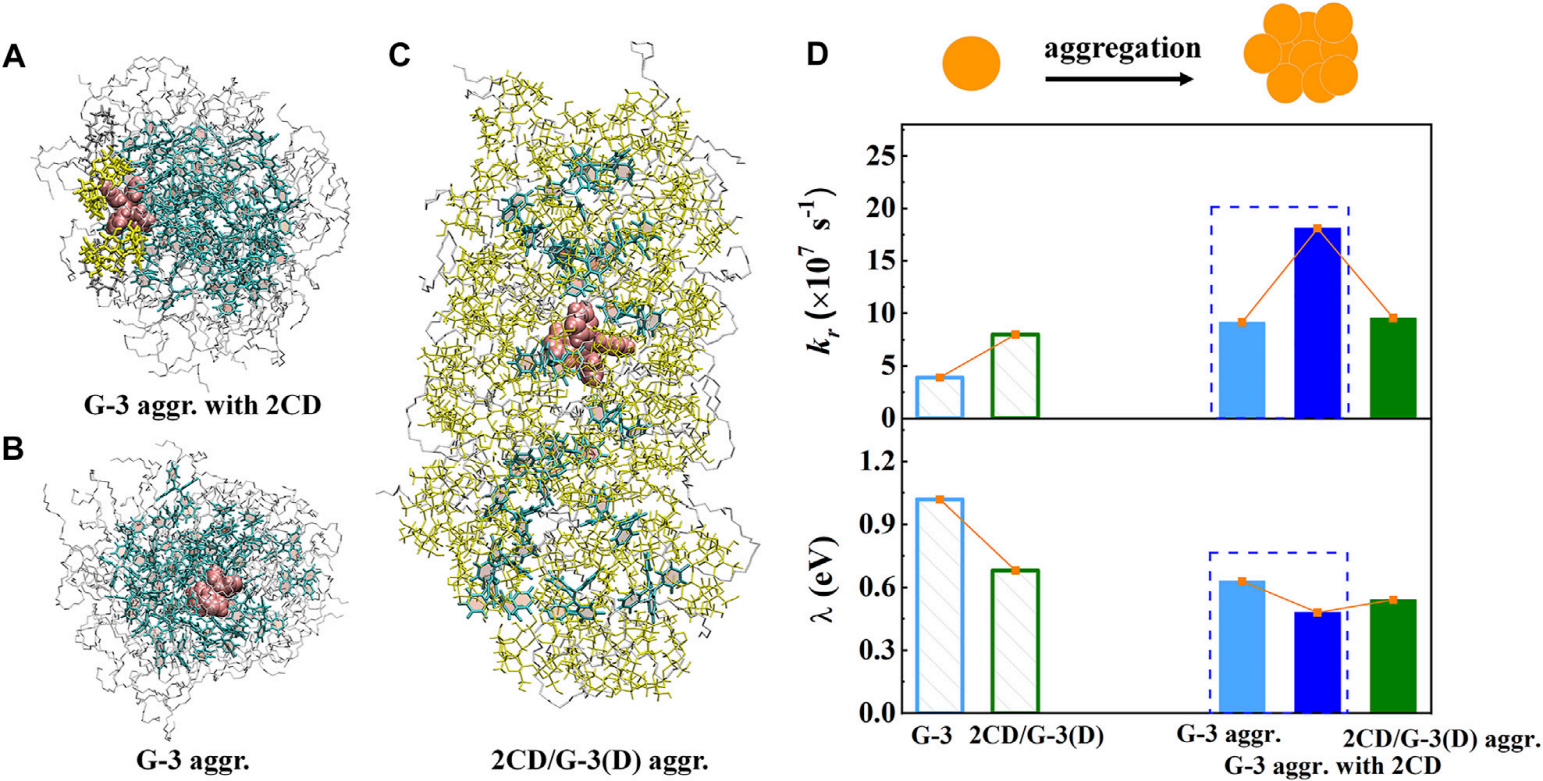
**(A–C)** The QM/MM models for the G-3 aggregate, G-3 aggregate with 2CD, and 2CD/G-3D aggregate, respectively. To be shown more clearly, we take “aggregate” as the abbreviation “aggr.” here. **(D)** The calculated *k*_r_ and *λ* of two monomers and three aggregates, respectively. (Reproduced with permission from Yang et al. (2021); Copyright 2021 The Royal Society of Chemistry).

Experimentally, a supramolecular host with a specific cavity can encapsulate proper-size AIEgens and form host–guest complexes, emitting fluorescence in the dispersed monomer (Liang et al., 2014; Song et al., 2014; Liang et al., 2016; Liow et al., 2017). However, the detailed structure–property relationship that determines the host–guest interaction–induced emission enhancement phenomenon remains elusive. A typical host molecule CD and a TPE derivative (G-3, Scheme 1) were taken as examples to study the influence of host–guest interactions on the photophysical properties of AIEgens by multiscale modeling protocol (Yang et al., 2021). MD simulations confirm that the host–guest inclusion complex 2CD/G-3(D) was formed by several cooperatively interplayed non-covalent interactions. On the one hand, the interior hydrophobic cavity of CD hosts one phenyl ring of the TPE moiety and partial PEG chain of the guest by the hydrophobic interaction. On the other hand, the exterior hydrophilic surfaces of CD fasten the PEG chain and adjacent phenyl rings of the TPE moiety of the guest by the intermolecular hydrogen bond and O-H^…^π interactions, respectively. Importantly, three representative aggregates: G-3 aggregate, G-3 aggregate with 2CD, and 2CD/G-3(D) aggregate were also simulated by MD simulations to consider the aggregation effect. The QM/MM models for all three kinds of aggregates were setup accordingly to further obtain the photophysical properties (Figures 5A–C). The *k*_r_ and *λ* are calculated and summarized in Figure 5D, where *λ* measures the extent of intramolecular electron–vibration coupling; the decrease in *λ* implies the sharp reduction of *k*_ic_. Introducing host–guest interaction, the fluorescent emission of the single inclusion complex 2CD/G-3(D) obviously increases relative to G-3 because of the slightly increased *k*_r_ and the sharply decreased *λ*. It is suggested that the host–guest interactions are responsible for hindering the non-radiative decay channel of the excited state energy of G-3. Upon aggregation, *k*_r_ of the G-3 aggregate and G-3 aggregate with 2CD are sharply boosted; at the same time, the corresponding *λ* is decreased, which is beneficial to the enhanced emission of aggregates. However, further increasing the concentration of CD is not conducive to luminescence because the high concentration of CD causes the disassembling of the 2CD/G-3(D) aggregate and the decrease of packing density; thus, the non-radiative decay channel is unblocked again. Therefore, the aggregation effect coupled with host–guest interactions in the G-3 aggregate with the 2CD system could significantly restrict the low-frequency rotational motions of phenyl rings and the C=C double bond twisting of the TPE moiety effectively; therefore the non-radiative decay channels of excited state energy in amphiphilic AIEgens are effectively blocked and finally enhances the fluorescent intensity.

### Organic Aggregates Involving Co-Crystallized Solvent Molecules

**FIGURE 6:**
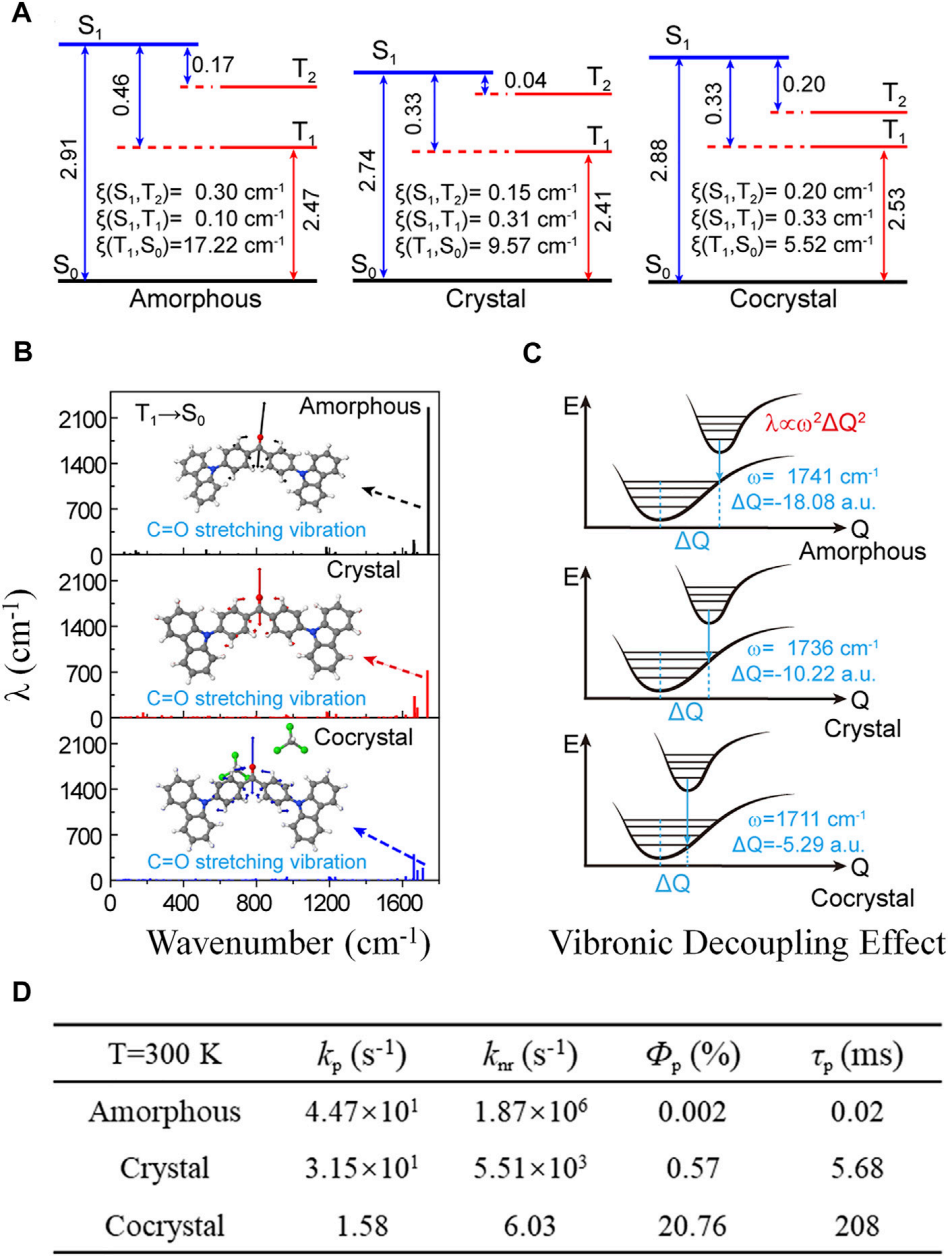
**(A,B)** Calculated spin-orbital coupling coefficients (*ξ*) and reorganization energies of low-lying excited states in amorphous, crystal, and cocrystal phases of Cz2BP. **(C)** Illustration of the vibronic decoupling effect of the electron and C=O stretching vibration. **(D)** Calculated key parameters (*k*_p_, *k*_nr_, *Ф*_p_ and τ_p_) of the exciton energy decay process in amorphous, crystal, and cocrystal. (Reproduced with permission from Ma et al. (2019); Copyright 2019 American Chemical Society).

The luminescent properties of organic molecules are highly morphology-dependent (Ma et al., 2016; Taylor and Wood, 2019; Fu et al., 2021). 4,4′-bis(9H-carbazol-9-yl)-methanone (Cz2BP, Scheme 1) was observed to emit room-temperature phosphorescence in a cocrystal consisting of chloroform but not in the amorphous nor the crystal phase (Li et al., 2015). The impact of the intermolecular hydrogen bond interactions on luminescent properties of Cz2BP was quantitatively investigated by the thermal vibration correlation function–coupled QM/MM calculations (Ma et al., 2019). It is found that compared with amorphous aggregate and crystal, the strong intermolecular hydrogen bond (C=O^…^H−C) between Cz2BP and chloroform in cocrystal makes the densest molecular packing and effectively decouple the vibronic effect. For the T_1_ state, responsible for phosphorescence, its relative compositions of (n, π*) and (π, π*) and the spin-orbital coupling coefficients (*ξ*) strongly depend on the aggregation. From amorphous to crystal to cocrystal, the *ξ*(T_1_→S_0_) decreases from 17.22, 9.57 to 5.52 cm^−1^, while the corresponding (π, π*) components of the T_1_ state are 59.8, 88.6, and 94.6%, respectively (Figure 6A). The electron–vibration coupling analysis (Figure 6B) indicates that the λ is dominated by high-frequency modes, including the stretching vibration of the C=O bond and the breathing vibration of benzene and carbazole units. In particular, the C=O stretching vibration (ω) is drastically reduced from 1,888.46 to 717.24 to 186.67 cm^−1^ from amorphous to crystal to cocrystal, and the corresponding normal-mode displacement ΔQ is also regularly shortened, consecutively (Figure 6C). The quantum efficiency (*Ф*_P_) of RTP is quantitatively analyzed by the equation *Ф*_P_ ≈ *k*_p_/(*k*_p_ + *k*_nr_); the results indicate that the calculated *k*_p_ of T_1_→S_0_ decreases about one order of magnitude in cocrystal, more importantly, *k*_nr_ of T_1_→S_0_ is largely reduced by 3−6 orders of magnitude from 1.87 × 10^6^ to 5.51 × 10^3^ s^−1^ to 6.03 s^−1^, leading to an efficient *Ф*_P_ (20.76%) in cocrystal relative to the extremely low *Ф*_P_ in the amorphous and crystal, and finally inducing a bright and long-lived RTP (Figure 6D). Therefore, both the decreased *ξ*(T_1_, S_0_) and the decreased *λ* of the C=O stretching vibration contribute to the sharply decreased *k*_nr_, but the decoupling of the electron vibration from C=O plays the primary role in decreasing of *k*_nr_.

### Organic Aggregates Doping Trace Amounts of Host Molecules

**FIGURE 7:**
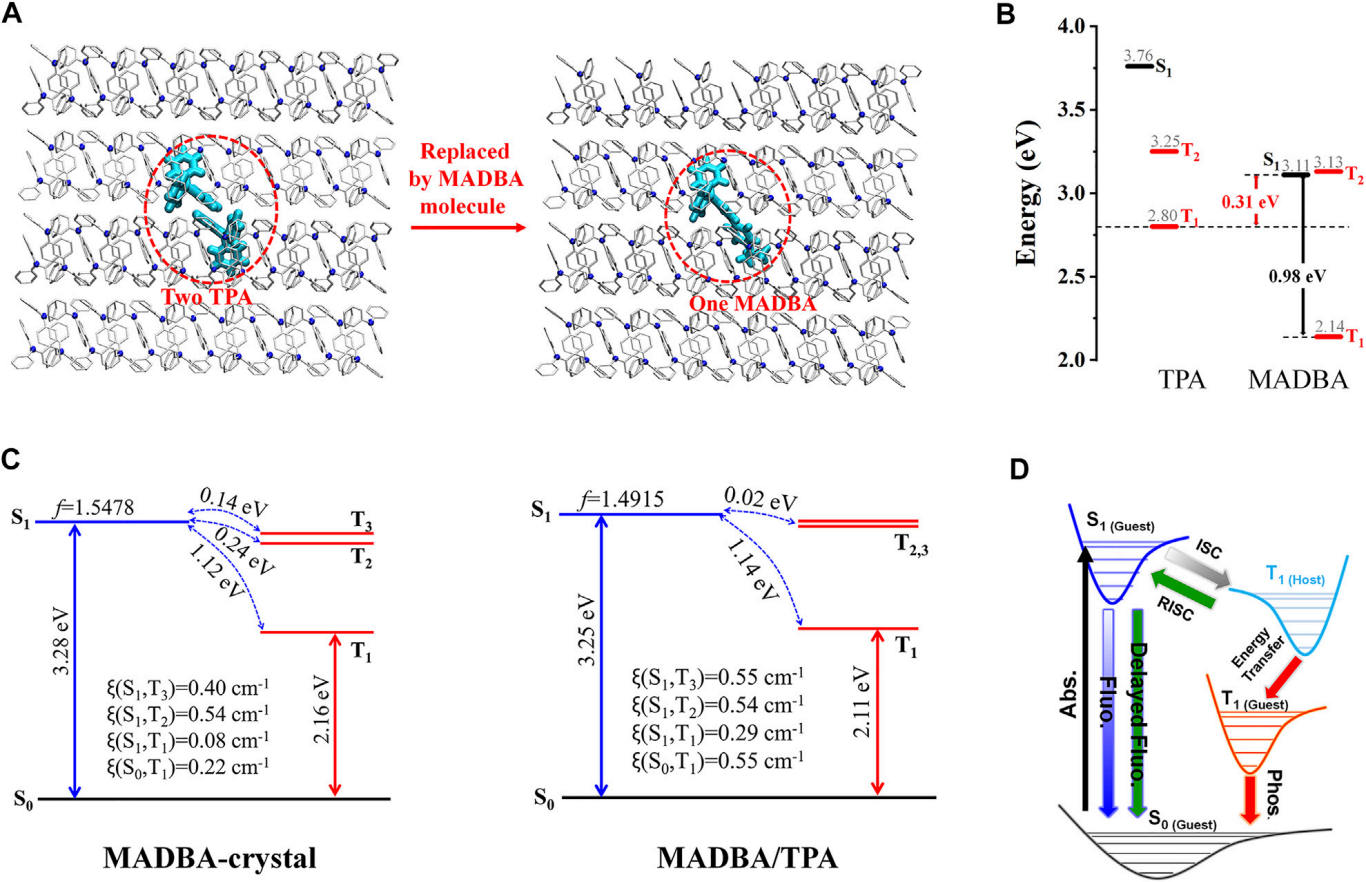
**(A)** Model setup of the doped system. **(B)** The energy levels of the host TPA and guest MADBA. **(C)** The calculated energy level diagram, spin-orbit couplings (*ξ*), and the oscillator strengths for MADBA crystal and the doped system. **(D)** Proposed transfer pathway between the guest and host after doping. (Reproduced with permission from Lei et al. (2021) Copyright 2021 The Royal Society of Chemistry).

Although a variety of doped organic systems with room temperature phosphorescence have been reported (Chen et al., 2021; Ning et al., 2021; Yan et al., 2021), the specific configuration and molecular packing of the guest molecules in the host matrix are still unknown. For example, we recently found that pure TPA crystalline powder only exhibits weak fluorescence, while the doping TPA matrix with no more than 0.1% guest molecule (MADBA, Scheme 1) simultaneously shows strong fluorescence, thermally activated delayed fluorescence, and efficient room temperature phosphorescence (Lei et al., 2021). We further setup a MADBA/TPA doping model with the molar ratio of 1: 190 by replacing two TPA molecules with a single large-sized MADBA molecule (Figure 7A). Then, MD simulations were performed to simulate the doped configurations and spatial molecular packing of the guest molecule in the host TPA matrix. To the best of our knowledge, this is the first use of MD simulations to study doped materials. The QM/MM model was further setup based on the equilibrated MD conformation of the doped system to study the photophysical properties. Since the larger volume of MADBA than that of the TPA molecule, the intermolecular distances between MADBA and surrounding TPA molecules are reduced with the intermolecular C–H^…^π interactions enhanced, indicating a more rigid environment of MADBA in the doping system. After doping, the structural changes of MADBA from S_0_ to T_1_ in the doped state become smaller than those in the solution state. In addition, it is found that the energy gap (ΔE_ST_) between S_1_ and T_1_ of MADBA is 0.98 eV, which is not facilitating the intersystem crossing (ISC) process (Figure 7B). The host TPA with a similar molecular structure shows higher T_1_ energy (2.80 eV) than MADBA; therefore, the T_1_ of TPA could act as a bridge to narrow the ΔE_ST_ from 0.98 to 0.31 eV to facilitate the ISC process of MADBA, namely, the intermediate T_1_ of TPA is beneficial for the energy transfer from the T_1_ of the host TPA to guest MADBA. Compared to pure MADBA crystal, the smaller ΔE_ST_ and larger *ξ* between S_1_ and low-lying triplet states also support the easier ISC process in the doped MADBA/TPA system than that in the pure MADBA crystal (Figure 7C). Therefore, matching the energy levels between host and guest molecules could effectively bridge the energy transfer process of the low-lying triplet states and facilitate the ISC process, which will make room temperature phosphorescence more efficient in the doping system (Figure 7D). Therefore, doping trace amounts of MADBA is beneficial to promote ISC of excitons, thereby leading to phosphorescence emission in the host matrix.

## Conclusion and Outlook

In this review, we summarize recent works on studying the structure–property relationships of organic aggregates at different aggregated states using multiscale modeling protocol, combining the molecular dynamics (MD) simulations and quantum mechanics/molecular mechanics (QM/MM) calculations. We conclude that 1) the FQE of a nano-sized aggregate is size-independent, and the embedded molecules dominate the fluorescent intensity of amorphous aggregates; there exists a linear relationship between the fluorescent intensity and aggregate size; 2) the dense molecular packing of non-typical AIEgens (annulene-based eight-member ring COTh) at the crystalline state can effectively suppress the aromatic reversal process and block the non-radiative decay channels, leading to the fluorescent emission in the crystal; 3) for the supramolecular host–guest complex, the aggregation effect coupled with non-covalent interaction–induced host–guest interactions can significantly retard the non-radiative decay channels of excited state energy and make the supramolecular host–guest complex emit light at both monomer and aggregated states; 4) host and guest molecules could effectively bridge the energy transfer process of the low-lying triplet states and facilitate the ISC process, thereby leading to phosphorescence emission. It is obvious that the multiscale modeling approach combining MD simulations and QM/MM calculations is applicable to simulate the structure–property relationship of complex systems in experiments and provide a direct explanation for the complex experimental phenomenon.

It is still a great challenge for simulating the conformations and photophysical properties of organic aggregates at various biological environments (such as the lipid membrane, lipid droplet, mitochondria, and so on) and providing a useful clue in the rational design of organic luminescent materials for bio-imaging and multi-modality theranostics. The influence of various external forces (shear, grinding, or hydrostatic pressure) on the photophysical properties of organic aggregates is still rarely investigated. In addition, the electron–density change in MM polarization of the QM/MM model also needs to be considered. And, only considering one QM molecule sometimes is not enough for systems with charge transfer or exciton interactions. Therefore, there is still a longstanding challenge for us, and we are actively addressing them.
